# Gel permeation chromatography process for highly oriented Cs_3_Cu_2_I_5_ nanocrystal film

**DOI:** 10.1038/s41598-022-08760-6

**Published:** 2022-03-17

**Authors:** Yu-Hong Cheng, Rikuo Suzuki, Narumi Shinotsuka, Hinako Ebe, Naoaki Oshita, Ryohei Yamakado, Takayuki Chiba, Akito Masuhara, Junji Kido

**Affiliations:** 1 Graduate School of Organic Materials Science, Yamagata University, 4-3-16 Jonan, Yonezawa, Yamagata 992-8510 Japan; 2 Graduate School of Science and Engineering, Yamagata University, 4-3-16 Jonan, Yonezawa, Yamagata 992-8510 Japan

**Keywords:** Nanoparticles, Quantum dots

## Abstract

The emergence of green materials has attracted considerable attention in the field of optoelectronics. Copper-based lead-free metal halide (with a near-unity quantum yield) obtained from Cs_3_Cu_2_I_5_ nanocrystals (NCs) can exhibit blue emission with a wavelength of 440 nm and provide outstanding stability for various applications. However, in practical applications, colloidal dispersion purity and film quality are inadequate toward a high-performance device. In this study, antisolvent-free gel permeation chromatography is used to purify Cs_3_Cu_2_I_5_ NCs. The purified Cs_3_Cu_2_I_5_ NCs exhibit a high photoluminescent quantum yield and provide a highly oriented single-crystal film. Density functional theory calculation results indicate that the iodide-rich surface in the NCs makes them highly stable. In addition, it has been demonstrated for the first time that the mixture of polymethyl methacrylate (PMMA) and Cs_3_Cu_2_I_5_ NCs has waterproofing capabilities. The composite film consisting of Cs_3_Cu_2_I_5_ NCs and PMMA can survive in water for several days. This result opens up more possibilities for the application of these green material.

## Introduction

Cesium lead halide perovskite nanocrystals (NCs) such as CsPbX_3_ (X = Cl, Br, I) have received tremendous attention for their use as optoelectronic materials in devices such as solar cells^1^, light-emitting diodes (LEDs)^2^, photodetectors^3,4^, and lasers^5^ owing to their outstanding optical properties: wide light absorption, high photoluminescence quantum yield (PLQY), and narrow emission spectra with tunable emission peaks. In spite of these advantages, the toxicity of lead and the poor stability at the B site of cesium lead halide perovskite NCs have prevented them from being commercialized^6–8^. In recent years, lead-free perovskite-related NCs containing low-toxicity metals such as tin^9,10^, bismuth^11^, and antimony^12^ have attracted attention as promising light-emitting materials for use in LEDs^13^. However, some challenges still need to be overcome with regard to the use of low-toxicity metals in NCs. For instance, Sn^2^ is usually very unstable because of its easy oxidization to tetravalent Sn^4+^ in atmospheric air. In addition, Bi^3+^-based lead-free NCs exhibit a low PLQY owing to their fast trapping state^14^.

More recently, the use of copper(I)-based lead-free metal halide structure as green materials has been demonstrated in the field of optoelectronics^15–28^. The photoluminescence (PL) spectra of lead-free Cs_3_Cu_2_I_5_ exhibited a deep blue emission with a broad full width at half maximum (FWHM) and a large Stokes shift, which is attributed to the strong self-trapped emission effect^29^. In particular, lead-free Cs_3_Cu_2_I_5_ NCs have the potential to provide strong electron–phonon coupling and a large exciton binding energy, which improve the PL intensity^15^. These NCs can be synthesized via a hot-injection method under high-temperature reaction conditions^30–32^, or a ligand-assisted reprecipitation method under room-temperature reaction conditions^16^. However, these NCs require a purification process involving antisolvent reprecipitation, which commonly causes optical quenching and aggregation of NCs. Therefore, an antisolvent-free purification process is urgently needed to realize the application of these NCs in optoelectronic devices. In this context, we previously developed a gel permeation chromatography (GPC) process to remove impurities using only toluene as the nonpolar agent via only one purification cycle^33^. This antisolvent-free purification process has been investigated in the context of core–shell NCs, size selection^34,35^, and ligand-exchange^36,37^, but has rarely been mentioned in the context of cesium lead halides^38,39^.

In this work, we demonstrated the use of antisolvent-free GPC for purifying lead-free Cs_3_Cu_2_I_5_ NCs. The purification process completely eliminated impurities in the NCs without causing optical quenching or aggregation. The X-ray diffraction (XRD) patterns of the GPC-purified Cs_3_Cu_2_I_5_ NC film exhibited a highly crystalline orientation similar to that of its single-crystal phase. Density functional theory (DFT) calculation results showed that the surface energy of Cs_3_Cu_2_I_5_ was the lowest at (020) owing to the high phase stability in the film. The GPC purified Cs_3_Cu_2_I_5_ NCs could be easily spin coated onto the substrate and showed a high PLQY of 79.7% with a peak wavelength of 443 nm and an FWHM of 74.3 nm. In addition, we blended GPC-purified Cs_3_Cu_2_I_5_ NCs with polymethyl methacrylate (PMMA) to improve the environmental stability properties of the NCs, such as water and thermal resistance. The PMMA-encapsulated NC-based free-standing film could be used for down-conversion filtering applications. We believe that the antisolvent-free GPC purification process can accelerate the development of lead-free NCs.

## Results and discussions

**Figure 1:**
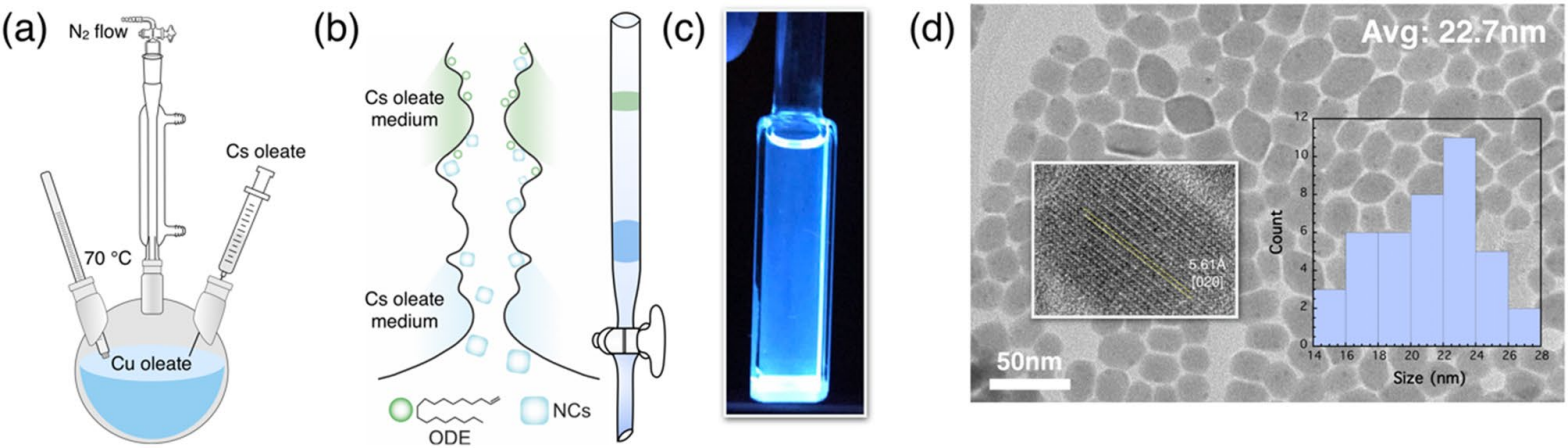
(**a**) Schematic of hot-injection process, (**b**) schematic of purification process, (**c**) image of GPC-purified colloid, and (**d**) TEM image of NCs after GPC purification with *d*-spacing and histogram of particle size.

Lead-free Cs_3_Cu_2_I_5_ NCs were synthesized via a previously reported hot-injection method with some modifications using cesium iodide (CsI) and copper iodide (CuI) as precursors^32^. We found that the synthesis conditions—ligand concentration and reaction temperature—were optimal for obtaining high-quality Cs_3_Cu_2_I_5_ NCs. CuI oleate was prepared by mixing CuI (6 mmol), oleic acid (OA, 6 mL), and oleylamine (OAM, 6 mL) with octadecene (ODE, 100 mL) in a three-neck flask under vacuum at 100 °C. Subsequently, Cs oleate was quickly injected into the CuI oleate solution at 70 °C (Fig. 1a). After 10 s, the reaction mixture was rapidly cooled in an ice bath. The as-synthesized Cs_3_Cu_2_I_5_ NCs were isolated via centrifugation at 10,000 rpm for 10 min, and then, the collected Cs_3_Cu_2_I_5_ NCs were redispersed in toluene, which was used as a nonpolar solvent. Cs_3_Cu_2_I_5_ NCs are very sensitive to polar antisolvents during the reprecipitation process. For instance, when ethyl acetate was used as an antisolvent in the reprecipitation of the NC colloid to remove impurities, the reprecipitated Cs_3_Cu_2_I_5_ NCs showed unfavorable crystal growth and a random crystal shape (Figure S1, supplementary information).

Thus, we developed a GPC purification process for Cs_3_Cu_2_I_5_ NCs to remove impurities and form a high-quality film. The major benefit of GPC purification is that only toluene is used as a developing solvent in this process, which can produce stable Cs_3_Cu_2_I_5_ NCs. The packing medium—polystyrene beads—is a porous and spherical ball, which enables the removal of small molecular hydrocarbon impurities such as ODE or unbound ligands, as shown in Fig. 1b. The Cs_3_Cu_2_I_5_ NC toluene colloid (3 mL) was injected into a polystyrene bead-packed column tube (see the Experimental section for details). Purified Cs_3_Cu_2_I_5_ NCs (1 mL) were obtained at a concentration of 10 mg mL^−1^ (Fig. 1c**)**. The Cs_3_Cu_2_I_5_ NCs had high colloidal stability, considering that the NC solution remained without causing precipitate at least a month. The quantified zeta potential both before and after GPC purification had positive valves: 18.2 and 15.6 mV, respectively. The formation of egg-like Cs_3_Cu_2_I_5_ NCs was confirmed by transmission electron microscopy (TEM) and histograms, as shown in Fig. 1d and Figure S2. The average sizes of the NCs before and after GPC purification were 20.7 and 22.7 nm, respectively. The GPC-purified NCs had an interplanar distance of 5.61 Å, which corresponded to the (020) crystal phase.

**Figure 2:**
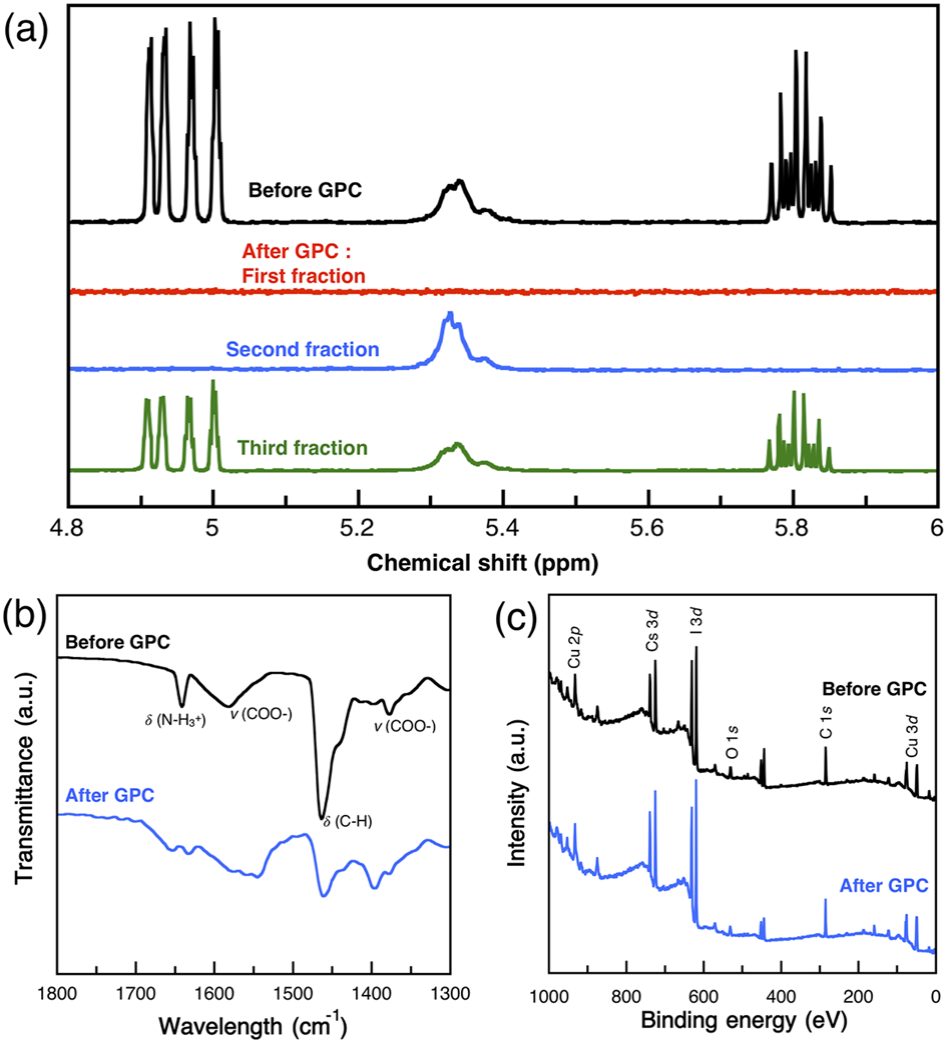
The surface analysis of (**a**) ^1^H NMR spectra of NCs collected from different fraction of GPC in chloroform-d1, (**b**) FTIR spectra, and (**c**) XPS spectra before and after GPC purification.

^1^H-NMR analysis was performed to determine residual impurities, as shown in Fig. 2a and Figure S3. The Cs_3_Cu_2_I_5_ NCs without GPC purification clearly showed alkene resonance related to the ODE at 5.7 to 5.9 ppm, which indicated that the purification was not completed; the OA and OAM related to the range at 5.2 to 5.4 ppm. We started the collection of samples when the Cs_3_Cu_2_I_5_ NCs was reached the exit of column. The position of Cs_3_Cu_2_I_5_ NCs was checked by UV lamp. In the first fraction, no resonances such as OA and OAM ligands, ODE were observed. Then, the NMR resonance of only surface ligands from Cs_3_Cu_2_I_5_ NCs without impurities peaks was observed for the second fraction. Finally, the third fraction showed both of surface ligands and impurities indicating the trapped ODE came together with resident Cs_3_Cu_2_I_5_ NCs. The surface ligand composition of the Cs_3_Cu_2_I_5_ NC film was confirmed by Fourier transform infrared spectroscopy (FTIR). The FTIR resonance showed the absent of OAM on Cs_3_Cu_2_I_5_ NCs surface showed in Fig. 2b. A study pointed out that the OA might tend to absorb onto the NCs, whereas the OAM was likely to desorb and leave the surface^40^. OAM is the primary capping ligand for lead based perovskite and the help of forming soluble molecular species of PbX_2_^41^. Therefore, OAM plays a role to form a soluble species of CuI during the Cu-oleate preparation. In addition, X-ray photoelectron spectroscopy (XPS) was used to elucidate the chemical composition of the Cs_3_Cu_2_I_5_ NCs (Fig. 2c and Figure S4). The estimated average atomic percentages of Cs, Cu, and I were 26.99%, 17.53%, and 55.47%, respectively, indicating a slightly halide-rich chemical composition. After the GPC process, the chemical composition of the purified NCs remained the same as that before the GPC process (Table S1, supplementary information).

**Figure 3:**
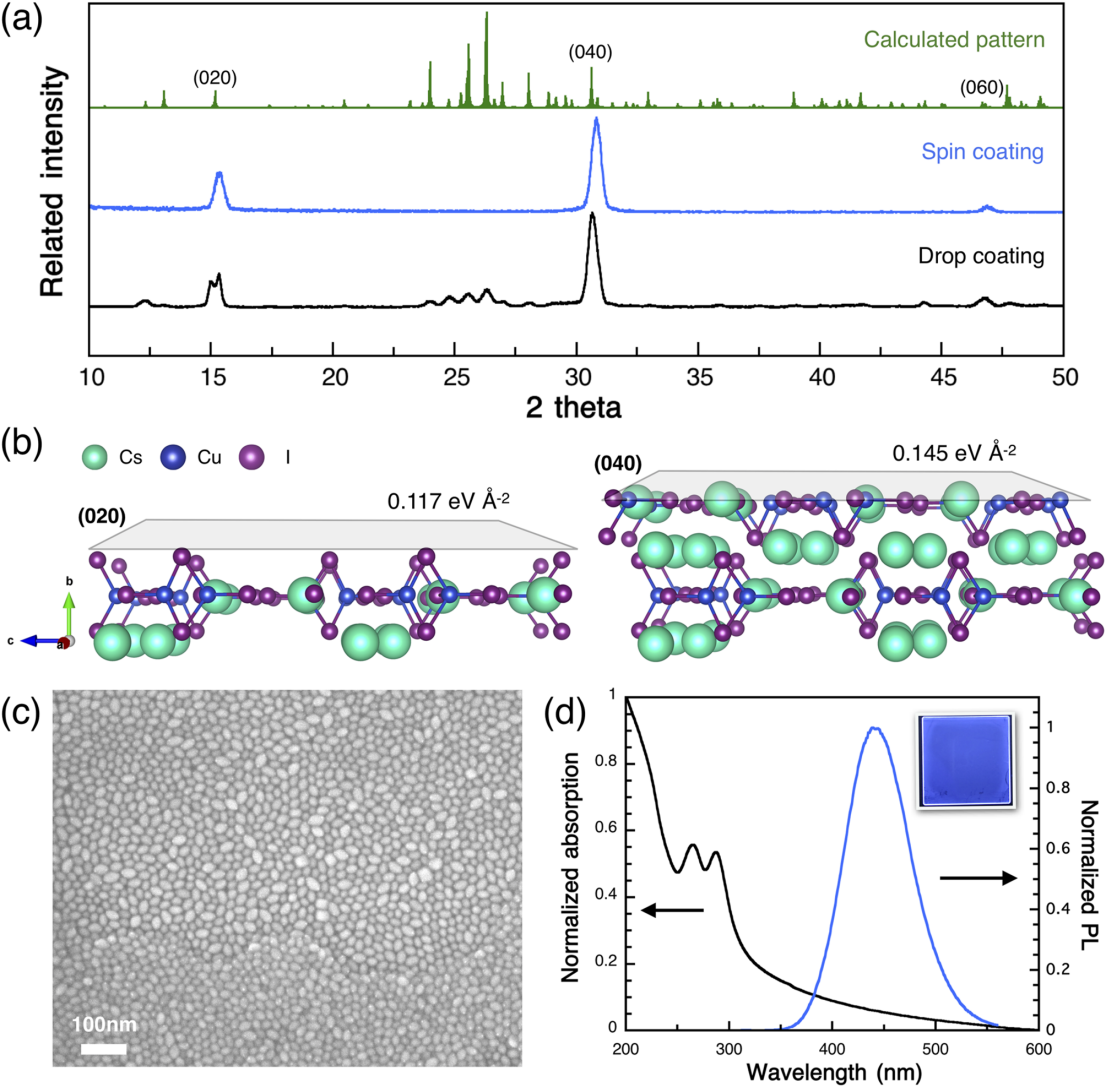
The structure and optical properties of the thin film. (**a**) XRD spectra of spin-coated and drop-coated films, (**b**) crystal structure with different surface directions, (**c**) SEM image of Cs_3_Cu_2_I_5_ NC neat film, and (**d**) UV–vis and PL spectra.

Cs_3_Cu_2_I_5_ single crystals were prepared from a saturated CsI and CuI precursor solution in dimethyl sulfoxide (DMSO), and then, single-crystal XRD (SXRD) analysis was performed. The obtained crystal data (Pnma, *a* = 10.1824(8) Å, *b* = 11.6655(11) Å, and *c* = 14.3687(12) Å) are consistent with those in previous reports (Table S2)^15,42^. On the other hand, the thin-film XRD analysis of the GPC-purified Cs_3_Cu_2_I_5_ NC film showed a simple crystal pattern with peaks at (020), (040), and (060) (Fig. 3a and Table S3). This diffraction pattern matched the calculated diffraction pattern obtained from the single-crystal structure determination. Interestingly, the spin-coated Cs_3_Cu_2_I_5_ NC film exhibited a highly oriented single-crystal arrangement in which the *b*-axis was vertical to the substrate, whereas the drop-coated film was randomly arranged. Drop casting was prepared by dropping NCs colloid onto 80℃ Si substrates. Therefore, the XRD peaks of drop coated NCs film were different from the spin coated film due to the random arrangement of Cs_3_Cu_2_I_5_ NCs. DFT calculation results showed that the surface energy of the (020) phase (0.117 eV Å^–2^) was lower than that of the (040) phase (0.145 eV Å^–2^), indicating that it was easier for the (020) phase than for the (040) phase to form on the surface (Fig. 3b and Figure S5)^40^. In the crystal model, (020) showed an iodide-rich surface, which was consistent with the XPS results.

Furthermore, the SEM image showed a close-packed thin film, which had egg-like shapes that tended to lie flat on the substrate (Fig. 3c). This unique shape is also considered as the factor of promoting the orientated film. Here, we have drop coated NCs colloid onto substrate at different temperatures, there is no obvious difference in the XRD pattern [Figure S6(a)]. On the other hand, the spin coated film had an extra centrifugal force which distinguished the difference in the diffraction pattern. A lower speed coated film showed a similar pattern with drop coated film. As increasing the spin speed, the XRD pattern tend to orientate at (020) and (040) phase [Figure S6(b)]. Therefore, we concluded that the promotion of the orientated film is attributed to the high surface energies, the egg-like shape, and centrifugal force by spin coating process.

The UV–vis absorption and PL spectra of the GPC-purified Cs_3_Cu_2_I_5_ NC films are shown in Fig. 3d. The optical band edge of the Cs_3_Cu_2_I_5_ NC film was at 316 nm, which corresponded to a bandgap of 3.92 eV. The PL spectra of the Cs_3_Cu_2_I_5_ NC film exhibited blue emission at 443 nm with an FWHM of 74.3 nm. The PLQY and PL decay time of the Cs_3_Cu_2_I_5_ NCs were 79.3% and 1.284 µs in the film and nearly 100% and 1.236 µs in the colloidal solution, respectively (Figure S7). It is worth mentioning that the PLQY and PL decay time of the colloid before and after GPC purification showed no obvious changes owing to the suppression of optical quenching. Moreover, we dried the colloid using a vacuum pump and redispersed it back into toluene. We found that the PLQY reduced by only approximately 2% without any change in the emission wavelength, which is unlikely to happen in conventional lead halide perovskite NCs (Figure S8). The excellent redispersibility indicates less intense aggregation, compared with a lead-based perovskite, owing to their iodide-rich surface and low surface energy. Therefore, for the first time, we succeeded in investigating the GPC purification of Cs_3_Cu_2_I_5_ NCs and their optical properties in the solid film state. The detailed optical properties of the Cs_3_Cu_2_I_5_ NCs are listed in Table S4.

**Figure 4:**
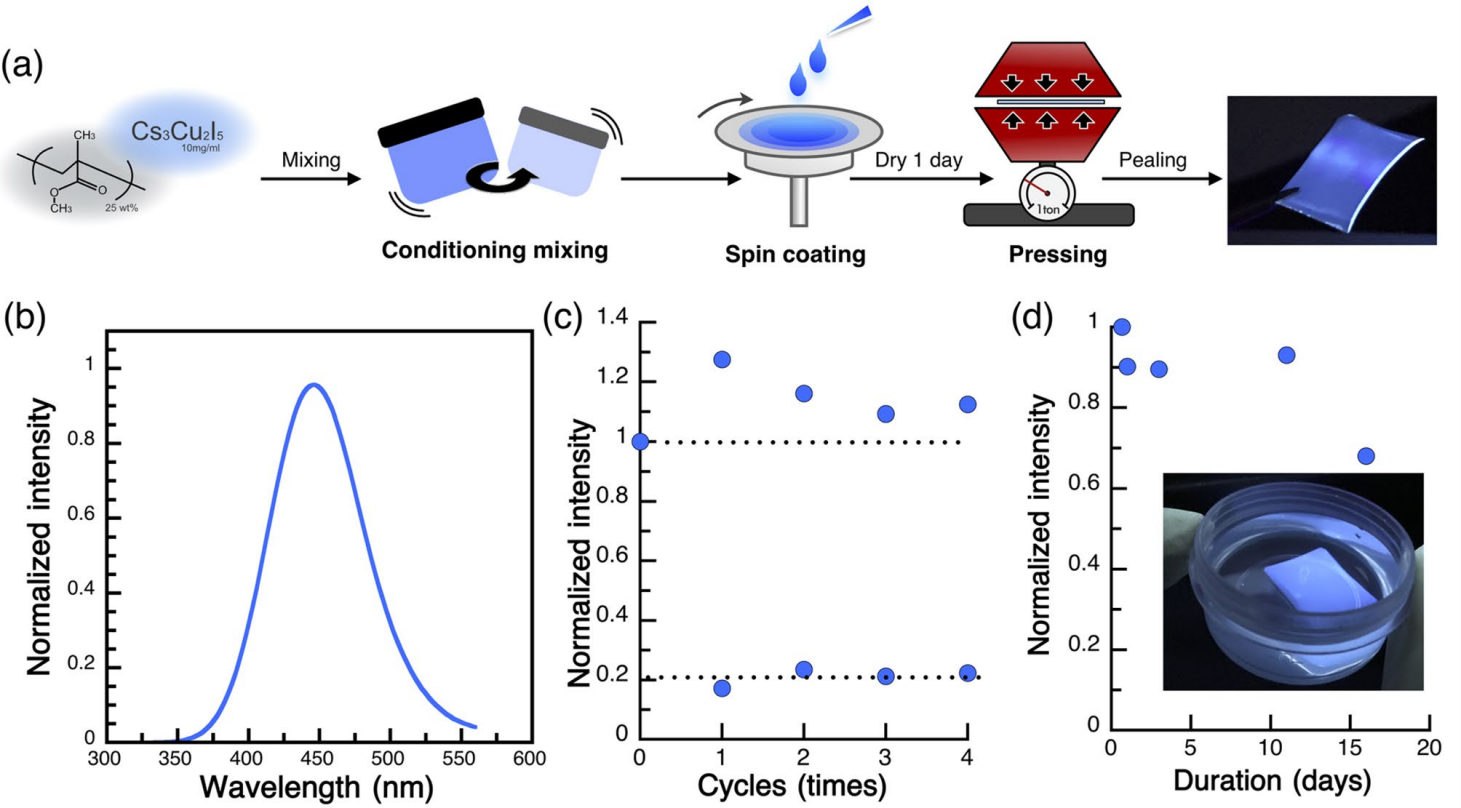
Polymer composite process and its properties. (**a**) Schematic of PMMA-blended NCs, (**b**) PL spectra of PMMA-blended NC film, (**c**) heating/cooling cycling PL measurements, and (**d**) water resistance test showing PL intensity with respect to days.

The improvement of environmental stability properties such as thermal stability, photostability, and water resistance is a key requirement for various optoelectronic applications. However, Cs_3_Cu_2_I_5_ NC films exhibit poor water resistance. We demonstrated for the first time that by blending Cs_3_Cu_2_I_5_ NCs with a PMMA polymer composite, a free-standing film could be obtained without a substrate and this film could be used for down-conversion filtering applications. The use of chemically stable polymers (PMMA and polystyrene) creates a framework for embedding conventional lead halide perovskite NCs; therefore, the film has improved stability against heat, light, and water owing to the hydrophobic protective property of polymers^43–45^. To prepare the free-standing Cs_3_Cu_2_I_5_ NCs blended with the PMMA film, a purified Cs_3_Cu_2_I_5_ NC colloidal solution (10 mg mL^−1^ in toluene) was mixed with a PMMA solution (25 wt% in chloroform) in a 1:3 volume ratio, and then, the mixture was kneaded for 2 min. After spin coating the blended film, compression treatment was carried out to produce flat films (Fig. 4a). The PL spectra of the blended film exhibited an emission wavelength of 443 nm with an FWHM of 74.9 nm and was identical to the PL spectra of the Cs_3_Cu_2_I_5_ NC neat film (Fig. 4b). The thermal stability of the blended film was tested in a temperature cycle between 25 and 100 °C, as shown in Fig. 4c. The PL intensity decreased as the temperature increased owing to the thermal quenching effect^41^. As the temperature returned to room temperature, the PL intensity also recovered to its initial value, indicating that there was no thermal degradation of the blended film. The blended film exhibited excellent water resistance and maintained the PL intensity even after 10 days at room temperature, as shown in Fig. 4d.

## Conclusion

In this study, polar-solvent-sensitive lead-free Cs_3_Cu_2_I_5_ NCs were obtained by GPC purification. The purified Cs_3_Cu_2_I_5_ NCs, which did not contain impurities, could form a pin-hole-free thin film. A polystyrene bead-packed column could provide a solution to other lead-free perovskites-related materials that might struggle with the purification process. The Cs_3_Cu_2_I_5_ NC film also showed a highly oriented crystal pattern, which was similar to its SXRD pattern, because of its iodide-rich surface and low surface energy. We also found that the oriented feature was further promoted by the unique egg-like sharp after spin coating. Finally, for the first time, we attempted to blend Cs_3_Cu_2_I_5_ NCs with PMMA to produce a free-standing film that could survive in water for at least 10 days. The optical properties of the PMMA-mixed Cs_3_Cu_2_I_5_ NCs remained the same as those of the unblended NCs, but the ability of the PMMA-mixed Cs_3_Cu_2_I_5_ NCs to resist water and humidity was better, which makes them suitable for down-conversion filtering applications.

## Materials and methods

### Materials

Cesium carbonate (Cs_2_CO_3_, 99.99%), octadecene (ODE, 90%), oleic acid (OA, 90%), oleylamine (OAM, 80%), were purchased from Sigma-Aldrich. Cesium iodide (CsI, 99.0%) was purchased from Tokyo Chemical Industry. Copper iodide (CuI, 99.0%) was purchased from Kanto Chemical. Poly(methyl methacrylate)(PMMA) pellet were purchased from Fujifilm Wako Pure Chemical. Beads were purchased from BIO-RAD (S-X1). All chemicals were used as received.

### Synthesis of Cs_3_Cu_2_I_5_ NCs

Pristine Cs_3_Cu_2_I_5_ NCs were synthesized via a modified hot-injection method^37^. To prepare Cs oleate, Cs_2_CO_3_ (273 mg, 0.84 mmol) and OA (0.95 mL, 2.7 mmol) were mixed with ODE (10 mL) in a three-neck flask, and the mixed solution was degassed under a vacuum environment at 120 °C for 1 h. To prepare CuI oleate, OA (6 mL, 17.1 mmol) and OAM (6 mL, 16.4 mmol) were mixed with ODE (100 mL) in a three-neck flask and degassed under vacuum at 120 °C for 1 h. After degassing, the flask was circulated with N_2_ flow. Then, CuI (1.1143 g, 6 mmol) was added to the flask at 100 °C until the entire CuI was dissolved. The flask was further cooled to 70 °C, and Cs oleate (8 mL) was quickly injected into it. After 10 s, the reaction was rapidly cooled in an ice bath. The products were isolated by air and centrifuged at 10,000 rpm for 10 min. The precipitates were then redispersed in toluene and recentrifuged at 10,000 rpm for 10 min to collect the supernatant. Finally, the colloid was filtered using a 0.22 µm PTFE filter.

### Synthesis of Cs_3_Cu_2_I_5_ single crystal

A Cs_3_Cu_2_I_5_ solution was prepared using DMSO under ambient conditions. DMSO was slowly added under vigorous stirring until its saturation state, where the concentration was around 0.75 M, and then, the solution was filtered using 0.22 µm PTFE syringe filters. The filtered solution was filled in a vial and covered with a paraffin film with a small hole. The vial was placed inside a bigger bottle filled with methanol below the vial, and then, the bottle was covered with a paraffin film. The bottle was then placed at room temperature. After 3 days, a single crystal was obtained.

### GPC purification process

Beads were allowed to swell overnight in a sample bottle. Then, the swollen beads were packed into a column with a height of approximately 17 cm and a diameter of 10 mm. Dehydrated toluene was made to continuously flow through the column until no free polystyrene was present in the eluent (tested by UV–vis spectroscopy). The Cs_3_Cu_2_I_5_ NC colloid (3 mL, obtained as described above) was mildly placed on the top of the column. Approximately 1 ml with concentration of 10 mg mL^−1^ purified dispersion can be obtained. Besides, samples collected at different elution times are used for characterization. All the steps were carried out inside a N_2_-filled glove box.

### Fabrication of blended NC PMMA film

A PMMA/chloroform solution (25 wt%) was prepared as follows. PMMA pellets (10 g) were dissolved in chloroform (20.1 mL) at 55 °C. The NC and PMMA/chloroform solutions (10 mg mL^−1^) were mixed in a 1:3 volume ratio; the mixture was then kneaded and defoamed. To fabricate the NC PMMA film, the NC PMMA mixed solution (1 mL) was spin coated onto a glass substrate at 50 rpm for 1000 s. Finally, the film was left in a darkroom for 1 day, and it was compressed under a weight of 1 ton for 1 min to form a flat surface.

### Characterization

Scanning electron microscope (SEM) imaging was took using JEOL JSM-6700F system operated at 15 kV, the samples were prepared by using ITO substrate. Transmission electron microscopy (TEM) analysis was performed using a JEOL JEM-2100F microscope operating at 200 kV. The NMR spectra were obtained using a JEOL 400 spectrometer operated at a ^1^H frequency of 500 MHz. FTIR was performed using a JASCO FT/IR-4700. The chemical compositions were determined by XPS (Thermo Fisher Scientific Theta probe). The single-crystal XRD data were collected using a Rigaku RAPID-II diffractometer with graphite monochromated Mo Kα radiation (λ = 0.71075 Å), while the out-of-plane XRD spectra were measured by a Rigaku SmartLab diffractometer using Cu-Ka raditation as the X-ray source; the sample was coated onto Si substrate. The photoluminescence spectra were measured using a HORIBA FluoroMax-2 luminescence spectrometer with a Xe lamp; PLQY were measured using a Hamamatsu C9920-01 integral sphere system at an exciton intensity of ~ 1 uW cm^−2^ with a Xe lamp, both PL and PLQY were measured by an excitation wavelength of 290 nm. Photoluminescence decay was determined using a Hamamatue C11367 Quantaurus-Tau system with an excitation wavelength of 280 nm. The UV–vis-absorption spectra were measured using a Shimadzu UV-3150 UV–vis-NIR spectrophotometer; all the optical measurements were performed by using quartz substrate. The water resistant was performed by immersed NCs + PMMA film into deionized water kept at room temperature. The zeta potential was performed using MALVERN Nano ZS.

## Supplementary Information

Supplementary Information.

## Data Availability

Materials and data are available.
