# Supplementary material for: Gel permeation chromatography process for highly oriented Cs3Cu2I5 nanocrystal film
Source: Sci Rep. 2022 Mar 17;12:4620. doi: 10.1038/s41598-022-08760-6 (PMC8931108; doi:10.1038/s41598-022-08760-6)
Supplement: Supplementary file 1 — Supplementary Information. [file 41598_2022_8760_MOESM1_ESM.docx]

Gel Permeation Chromatography Process for Highly Oriented Cs_3_Cu_2_I_5_ Nanocrystal Film

Yu-Hong Cheng^1^, Rikuo Suzuki^1^, Narumi Shinotsuka^1^, Hinako Ebe^1^, Naoaki Oshita^2^, Ryohei Yamakado^1^, Takayuki Chiba^1^ *, Akito Masuhara^2^, and Junji Kido^1^ *

1 Graduate School of Organic Materials Science, Yamagata University, 4-3-16 Jonan, Yonezawa, Yamagata 992-8510, Japan
2 Graduate School of Science and Engineering, Yamagata University, 4-3-16 Jonan, Yonezawa, Yamagata 992-8510, Japan
*Corresponding authors
Takayuki Chiba: [T-chiba@yz.yamagata-u.ac.jp](mailto:T-chiba@yz.yamagata-u.ac.jp) and Junji Kido: [kid@yz.yamagata-u.ac.jp](mailto:kid@yz.yamagata-u.ac.jp)

**DFT calculation:**

The crystal structure of Cs_3_Cu_2_I_5_ was obtained from SXRD spectra by Rietveld refinement. For slab systems, surface reconstruction and single-point calculations were performed, whereas for the bulk systems, single point calculations were performed at the ZORA-GGA-PBE/DZP theoretical level using the ADF BAND package. In the slab and bulk system, the cif files listed in a separate file were used. For the slab system, two layers of the bulk system were used in the (020) direction, where the surfaces are (020) and (040).

The surface energy was calculated from the following equation:

$\sigma=\frac{1}{A}(E_{slab}-nE_{bulk})$ (1)

where σ is the surface energy, A is the surface area of the slab on both sides, E_slab_ is the model of the slab system, n is a factor to match the number of atoms in the bulk system with the number of atoms in the slab system, and E_bulk_ is the energy of the bulk system. The calculation was based on the exact crystal model with infinite boundary of a-axis and c-axis shown in **Figure S5** ^1^**.**


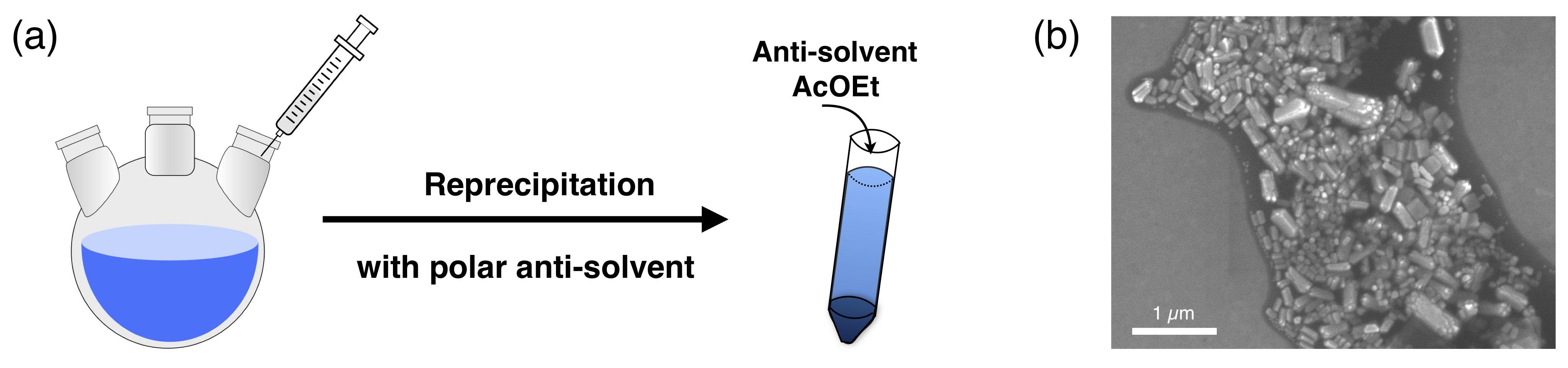


**Figure S1** (a) Schematic of hot-injection and typical purification process by adding anti-solvent to the Cs_3_Cu_2_I_5_ dispersion. (b) the SEM of the precipitate of anti-solvent treated dispersion.


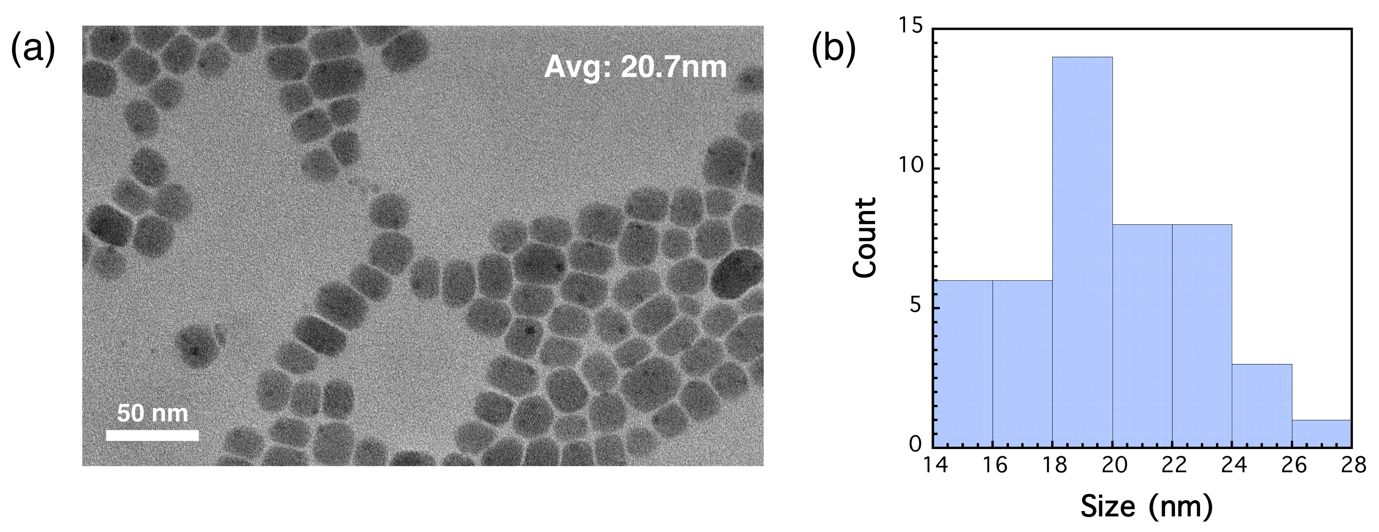


**Figure S2** (a) the TEM image of Cs_3_Cu_2_I_5_ NCs before processing GPC and its (b) size distribution.

.


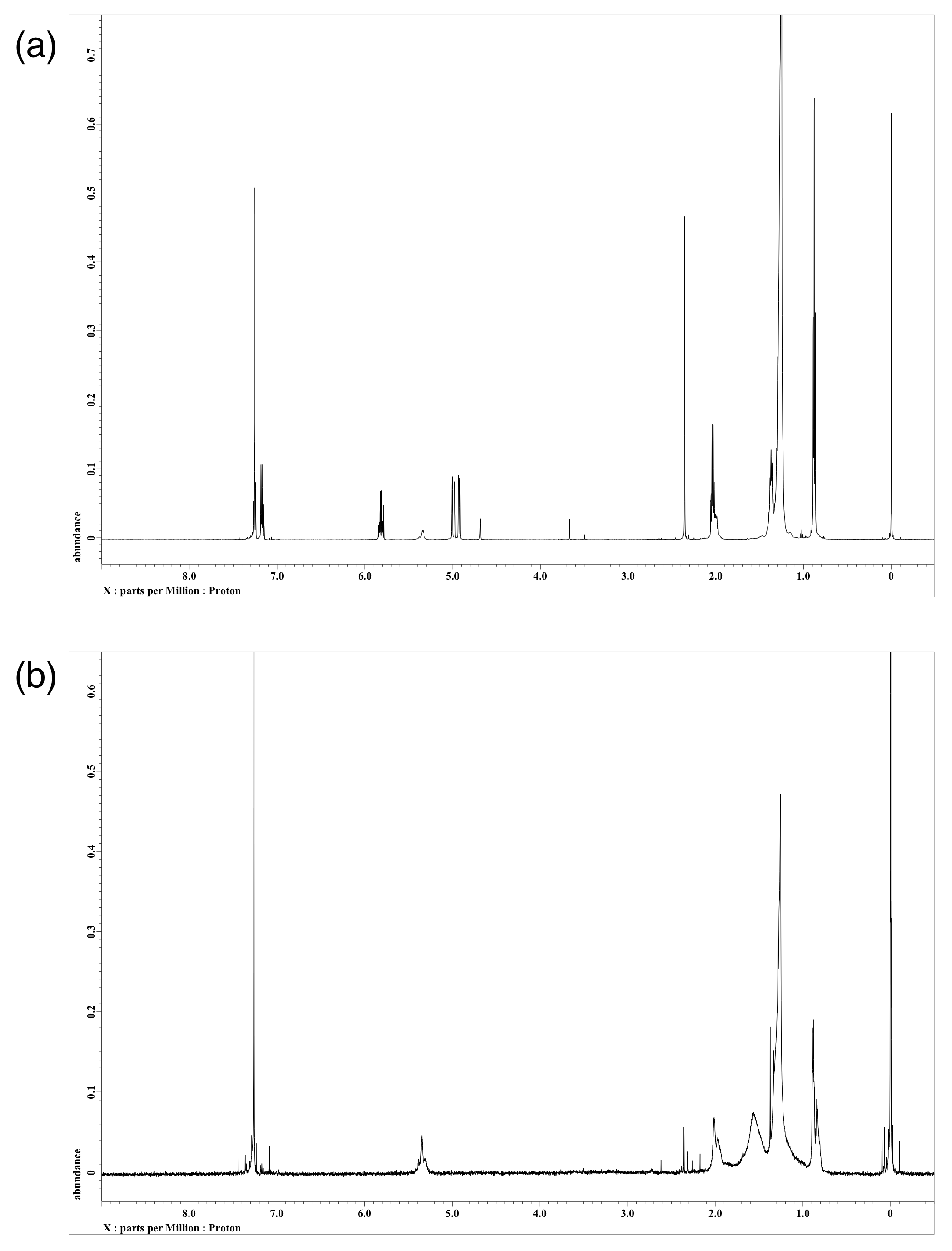


**Figure S3** ^1^H NMR full spectra of NCs in chloroform-d1(a) before GPC and (b) after GPC.

**Figure S4** The XPS spectra of Cs_3_Cu_2_I_5_ film prepared by the dispersion with or without GPC process: (a) Cs 3*d*, (b) Cu 3*p*, and (c) I 3*p*.


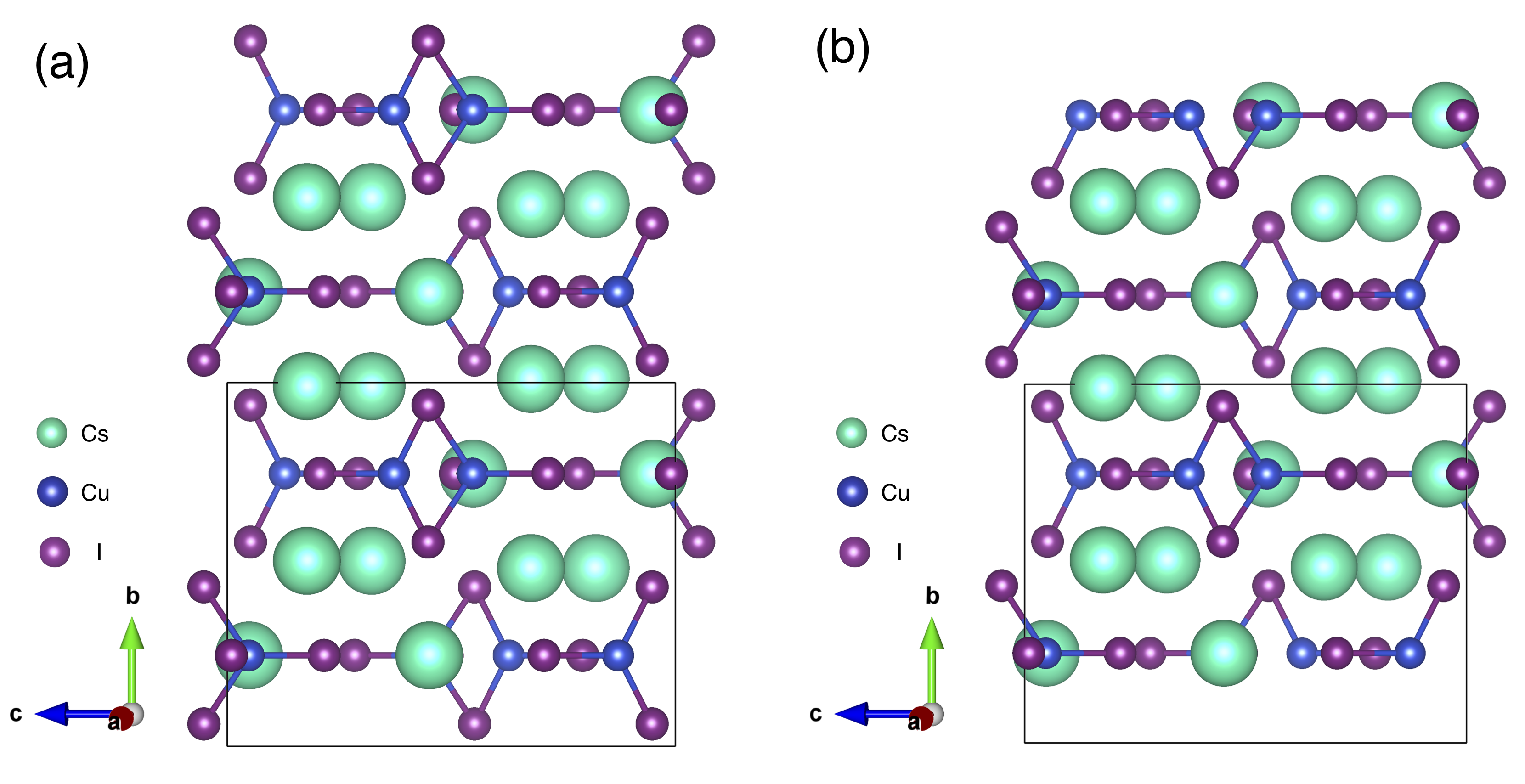


**Figure S5** The calculation is based on the model on a slab of (a) (020) phase and (b) (040) phase.


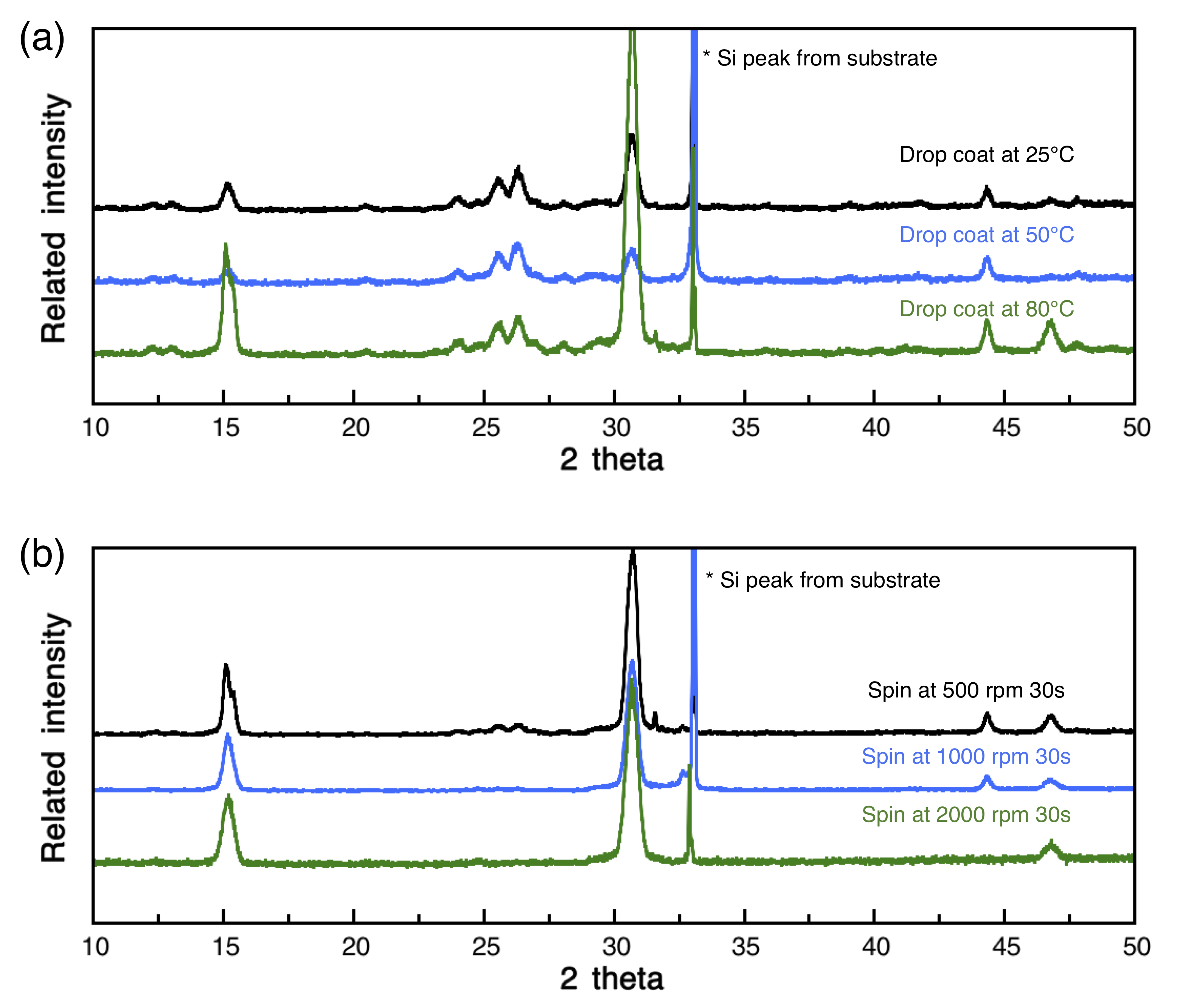


**Figure S6** XRD spectra of NCs prepared by different conditions (a) drop coated at different temperatures (b) spin coated by different spin speed.


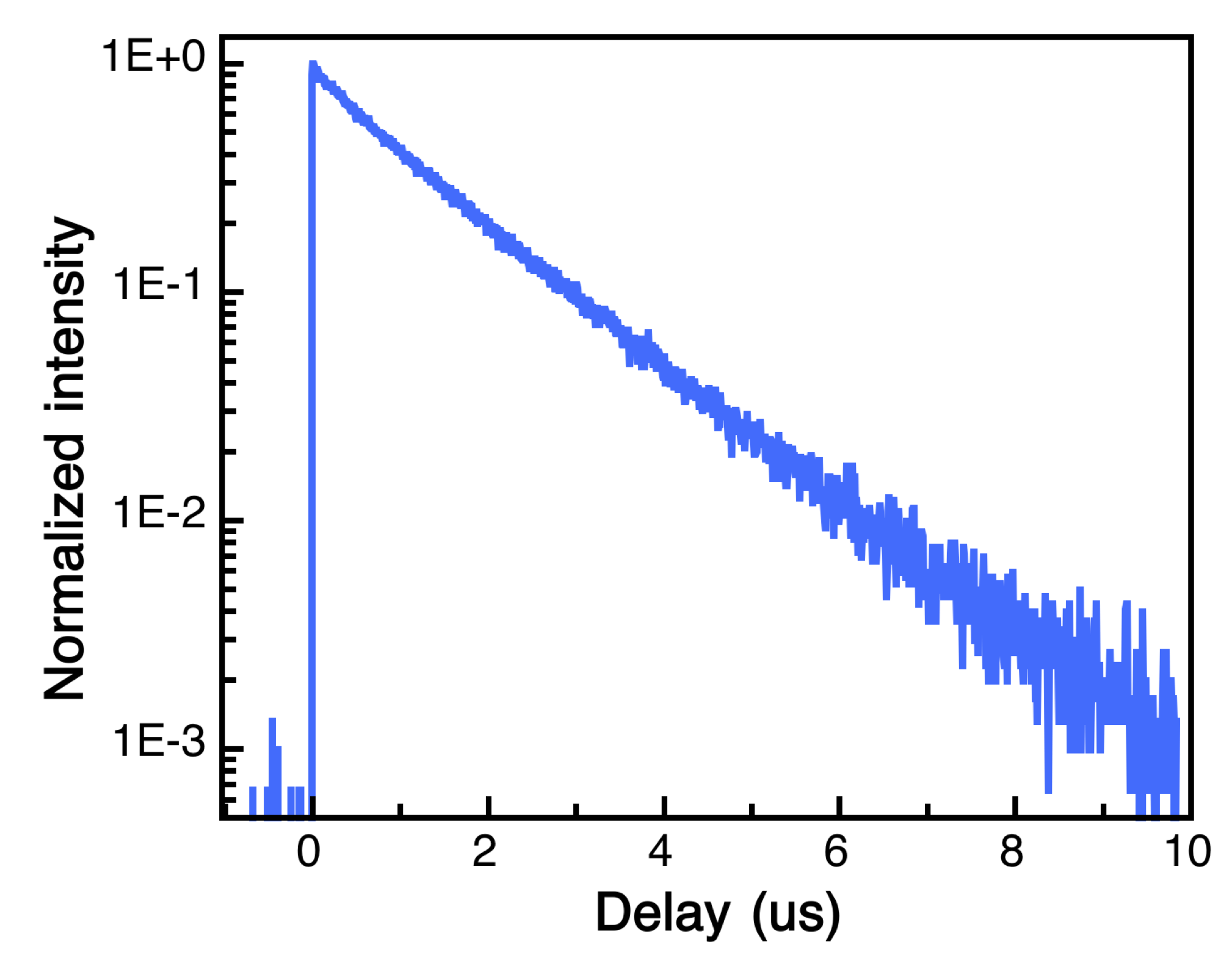


**Figure S7** The PL decay of Cs_3_Cu_2_I_5_ film.


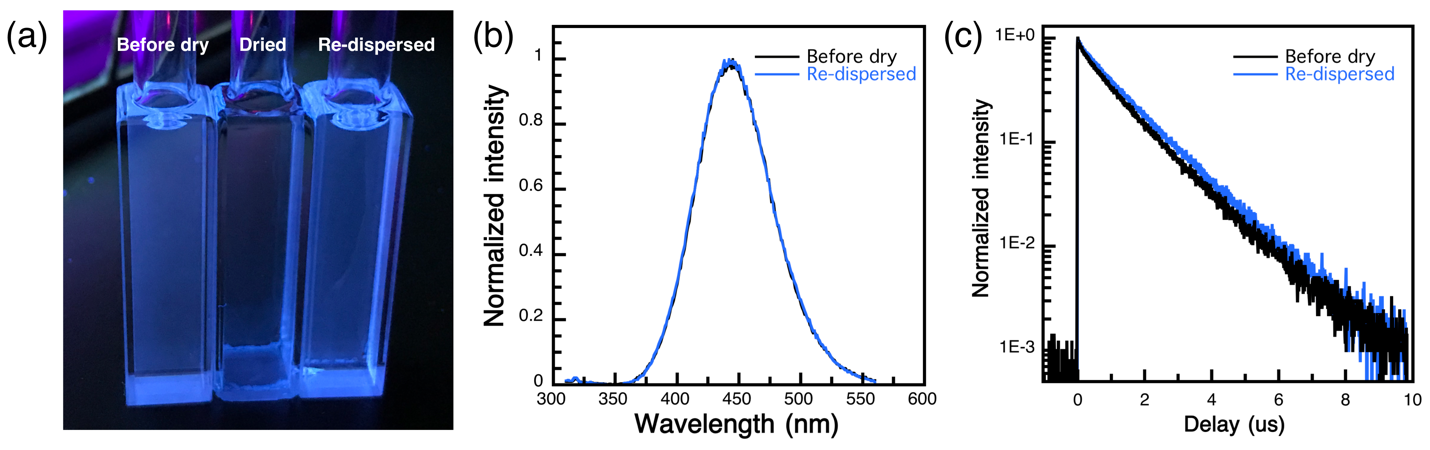


**Figure S8** (a) the photo of dispersion, dried cell, and re-dispersed solution in toluene, (b) the PL spectra, and the (c) PL decay spectra.

**Table S1** The XPS composition from before and after GPC NCs prepared by spin coating.

|  | **Cs** | **Cu** | **I** |
| --- | --- | --- | --- |
| **Before GPC** | 24.49 | 17.88 | 57.63 |
|  | 26.30 | 20.05 | 53.64 |
|  | 23.82 | 17.59 | 58.57 |
| **average** | **24.87** | **18.51** | **56.61** |
|  |  |  |  |
| **After GPC** | 26.38 | 17.32 | 56.29 |
|  | 27.99 | 17.04 | 54.98 |
|  | 26.61 | 18.24 | 55.15 |
| **average** | **26.99** | **17.53** | **55.47** |

**Table S2** Single crystal X-ray diffraction data for Cs_3_Cu_2_I_5_ NCs.

| Empirical formula | Cs_3_Cu_2_I_5_ |
| --- | --- |
| Temperature | 292(2) K |
| Source | Mo-Ka |
| Crystal system | Orthorhombic |
| Space group | *Pnma* |
| Unit cell dimensions | *a* = 10.1824(8) Å, alpha = 90 deg. |
|  | *b* = 11.6655(11) Å, beta = 90 deg. |
|  | *c* = 14.3687(12) Å, gamma = 90 deg. |
| Volume | 1706.8(3) Å^3^ |
| Crystal size | 0.500 × 0.460 × 0.310 mm |
| *Z* | 4 |
| *R*_1_ | 0.0434 |
| *wR*_2_ | 0.1120 |
| GOF | 1.126 |

**Table S3** The thin film XRD peak information and the comparison of calculated data.

| No. | Miller index | Calculated data *2θ* (deg.) | Thin film  *2θ* (deg.) | *d* (Å) | FWHM (deg.) |
| --- | --- | --- | --- | --- | --- |
| 1 | (020) | 15.178 | 15.320 | 5.779 | 0.396 |
| 2 | (040) | 30.630 | 30.799 | 2.9008 | 0.401 |
| 3 | (060) | 46.681 | 46.896 | 1.9358 | 0.454 |

**Table S4** The optical properties of each Cs_3_Cu_2_I_5_ in different forms.

| Samples | PL (nm) | FWHM (nm) | PLQY (%) | 𝛕_avg_ (µs) |
| --- | --- | --- | --- | --- |
| NCs colloid (Before GPC) | 444 | 73.1 | ~100 | 1.237 |
| NCs colloid (After GPC) | 443 | 73.5 | ~100 | 1.236 |
| NCs  re-dispersion | 442 | 72.4 | 98.3 | 1.175 |
| NCs film  (GPC purified) | 443 | 74.3 | 79.7 | 1.284 |
| NCs + PMMA | 443 | 74.9 | nd^(a)^ | 1.141 |

a) no data

1. K. Momma, F. Izumi, "VESTA 3 for three-dimensional visualization of crystal, volumetric and morphology data" J. Appl. Crystallogr. **44,** 1272 (2011).
